# Pharmacologic Inhibition of Host Phosphodiesterase-4 Improves Isoniazid-Mediated Clearance of *Mycobacterium tuberculosis*

**DOI:** 10.3389/fimmu.2016.00238

**Published:** 2016-06-17

**Authors:** Selvakumar Subbian, Mi-Sun Koo, Liana Tsenova, Vikram Khetani, Jerome B. Zeldis, Dorothy Fallows, Gilla Kaplan

**Affiliations:** ^1^The Public Health Research Institute (PHRI), Rutgers Biomedical and Health Sciences (RBHS), Rutgers University, Newark, NJ, USA; ^2^Office of Research Commercialization, Rutgers University, Piscataway, NJ, USA; ^3^Department of Biological Sciences, NYC College of Technology, Brooklyn, NY, USA; ^4^Celgene Corporation, Summit, NJ, USA; ^5^Bill and Melinda Gates Foundation, Seattle, WA, USA

**Keywords:** *Mycobacterium tuberculosis*, immune modulation, phosphodiesterase-4 inhibitor, mouse model, pulmonary tuberculosis, gene expression, host-directed therapy

## Abstract

The lengthy duration of multidrug therapy needed to cure tuberculosis (TB) poses significant challenges for global control of the disease. Moreover, chronic inflammation associated with TB leads to pulmonary damage that can remain even after successful cure. Thus, there is a great need for the development of effective shorter drug regimens to improve clinical outcome and strengthen TB control. Host-directed therapy (HDT) is emerging as a novel adjunctive strategy to enhance the efficacy and shorten the duration of TB treatment. Previously, we showed that the administration of CC-3052, a phosphodiesterase-4 inhibitor (PDE4i), reduced the host inflammatory response during *Mycobacterium tuberculosis* (Mtb) infection and improved the antimicrobial efficacy of isoniazid (INH) in both the mouse and rabbit models. In the present study, we evaluated the pharmacokinetics and explored the mechanism underlying the efficacy of a more potent PDE4i, CC-11050, as adjunct to INH treatment in a mouse model of pulmonary Mtb infection. Genome-wide lung transcriptome analysis confirmed the dampening of inflammation and associated network genes that we previously reported with CC-3052. Consistent with the reduction in inflammation, a significant improvement in Mtb control and pathology was observed in the lungs of mice treated with CC-11050 plus INH, compared to INH alone. This important confirmatory study will be used to help design upcoming human clinical trials with CC-11050 as an HDT for TB treatment.

## Introduction

Tuberculosis (TB), caused by *Mycobacterium tuberculosis* (Mtb), is a leading killer among infectious diseases, accounting for 1.5 million deaths and an estimated 9.6 million new cases in 2014.^1^ Standard short-course chemotherapy for pulmonary TB involves directly observed administration of four antibiotics, isoniazid (INH), rifampicin (RIF), ethambutol (EMB), and pyrazinamide (PZA) for 2 months, followed by INH and RIF therapy for 4 months (1). The long duration of treatment presents difficulties for case management and adherence, especially among patients from the poverty-stricken communities that are most affected. Inadequate therapy or treatment interruptions greatly elevate the risks of relapse and acquisition of drug resistance, thereby increasing the potential for further transmission and limiting therapeutic options for the patient. Moreover, residual lung damage and an elevated risk of reinfection can remain even after successful cure of TB (2–4). To overcome these problems, there is a great need for the development of effective shorter drug regimens to improve clinical outcome and enhance TB control. However, thus far, efforts to shorten TB chemotherapy have proven unsuccessful (5). This was most recently demonstrated in a series of randomized controlled clinical trials, showing that substitution of INH or EMB by one of the newer fluoroquinolones in a short-course regimen administered for 4 months led to an increased incidence of relapse, compared to the standard 6-month drug regimen (6).

Host-directed therapy (HDT) as an adjunct to conventional antibiotic regimens is emerging as a novel strategy for shortening the duration of TB therapy (7, 8). An important advantage of this approach is that Mtb cannot develop resistance to drugs that target host cell functions. Evidence from both *in vitro* and *in vivo* studies suggests that the extended duration of TB therapy necessary to achieve cure may be explained by an ability of the bacilli to shift to a slow or non-replicating state, in which they are not responsive to standard doses of the currently available antibiotics (5, 9). Non-replicating persistence of Mtb has been associated with diverse environmental pressures exerted by the host immune response, including hypoxia and the production of reactive nitrogen intermediates, which are capable of driving the bacilli toward a dormant phenotype *in vitro* (10).

Based on these reports, we hypothesized that dampening the inflammatory response, which is associated with macrophage activation and the environmental pressure on intracellular Mtb, through the use of adjunctive HDT, could render the bacilli more vulnerable to antibiotic-mediated killing. We have demonstrated the value of this approach by showing, in both the mouse and rabbit models, that adjunctive treatment with the small molecule phosphodiesterase-4 inhibitor (PDE4i), CC-3052, reduced the overt inflammatory response during Mtb infection and significantly improved INH-mediated bacillary clearance from the lungs (11–13). While the PDE4i alone had no impact on the growth of Mtb *in vitro*, within the lungs of CC-3052-treated animals, we observed a significant reduction in the expression of several Mtb stress response genes that are associated with dormancy (12). In addition, we noted changes in the pathologic manifestations of lung granulomas in both mice and rabbits in response to CC-3052. The reduction in pathology induced by PDE4 inhibition in the Mtb-infected lungs indicates that this treatment strategy may help to limit pulmonary damage for improved clinical outcome in TB patients.

The present study was undertaken to compare the impact of CC-3052 in the murine TB model with a second PDE4i (CC-11050), in preparation for a clinical trial of the latter drug in TB patients. CC-11050 shows improved stability over CC-3052, has demonstrated safety in humans, and has progressed to a phase II clinical trial for treatment of lupus erythematosus and a phase I trial in HIV-infected adults on antiretroviral therapy. To better understand the underlying molecular events associated with PDE4 inhibition during TB chemotherapy, we recently studied CC-11050 as an adjunct to INH treatment in the rabbit model of pulmonary TB. We showed that CC-11050 reduced inflammation and pathology in the lungs and increased the antibiotic responsiveness of the infecting Mtb (14). Here, we describe the pharmacokinetics of CC-11050 in mice and show that the drug reduces the inflammatory response and improves INH-mediated bacillary clearance from the lungs. Taken together, the results of our studies indicate that CC-11050 is a promising candidate for HDT that may shorten the duration of treatment and improve clinical outcome in patients with pulmonary TB.

## Materials and Methods

### Bacterial Growth and Chemicals

*Mycobacterium tuberculosis* CDC1551 was grown in Middlebrook 7H9 media (BD Biosciences, MD, USA) as described in Ref. (11). CC-11050 was provided by Celgene Corporation, Summit, NJ, USA. All other chemicals were purchased from Sigma (Sigma–Aldrich, MO, USA), unless stated otherwise.

### Animal Welfare and Ethical Statement

Eight-week-old female B6D2F1 mice (The Jackson Laboratory, Bar Harbor, ME, USA) were used in this study. All animal procedures, including aerosol infection, oral gavage treatment, postinfection/treatment monitoring, were performed following standard protocols approved by the Institutional Animal Care and Use Committee (IACUC) of Rutgers University and were in compliance with institutional and governmental guidelines regulating the care and use of laboratory animals in experimental research.

### Structure and *In Vivo* Pharmacokinetics of CC-11050

The PDE4i, CC-11050 (N-[2-[(1S)-1-(3-ethoxy-4-methoxyphenyl)-2-(methylsulfonyl) ethyl]-2,3-dihydro-3-oxo-1H-isoindol-4-yl]-(9Cl) cyclopropanecarboxamide) has an empirical formula of C_24_H_28_N_2_O_6_S with one chiral center (Figure S1A in Supplementary Material). Uninfected mice (*n* = 3 per group) were treated with 5, 25, or 50 mg/kg of CC-11050 by oral gavage. In another experiment, mice (*n* = 3) were treated with 50 mg/kg of CC-11050 plus 50 mg/kg of INH. A group of mice (*n* = 3) without treatment were included as negative control. Venous blood was collected at 1, 2, 5, 8, and 24 h after a single gavage administration of CC-11050, with or without INH. Time-matched blood samples were also collected from untreated mice. The animals were gavaged after overnight fasting and fed soon after gavage. Plasma was collected by centrifugation of blood samples at 1,100× *g* for 10 min at 10°C and used for LC-MS/MS analysis.

The standard curve and quality control for CC-11050 were prepared in mouse plasma diluted 1:1 with Sorenson’s Citrate Buffer (25 mM; pH 1.5). Internal standard was prepared with ^13^CD_3_-CC-11050 and mixed with the standard, quality control, or test samples at 4:1:1 (^13^CD_3_-CC-11050:mouse plasma:Sorenson’s Citrate Buffer, vol/vol/vol) ratio. The mix was centrifuged at 4,000 rpm for 10 min at room temperature, and the supernatant was transferred to a 96-well assay plate. LC-MS/MS was performed using a Sciex API 4000 triple quadrupole mass spectrometer (Sciex, Division of MDS Inc., Canada) coupled to a Shimadzu HPLC system (Shimadzu Scientific Instruments, MD, USA). A Phenomenex Gemini column (5 μm, 2.0 mm × 50 mm; Phenomenex, CA, USA) was used for chromatography with 0.1% formic acid in water (mobile phase A) and 0.1% formic acid in acetonitrile (mobile phase B). The parameters used were 0 min for 10%B, 0.0–2.0 min for 10–75%B, 2.1–3.0 min for 95%B, and 3.05–4.0 min for 10%B. For mass spectrometric detection, a positive ion mode with turbo spray (ion source temperature of 450°C) and a dwell time of 100 ms was used. Analytes were quantified using multiple reactions monitoring (MRM) at the following transitions: mass-to-charge ratio 473.1–178.1 and 477.1–182.1 *m*/*z* for CC-11050 and its internal standard, respectively. Peak area ratios of the analyte to its internal standard versus analyte concentration, represented as quadratic regression plots, were derived with 1/*x*^2^ weighting. Watson LIMS™ software was used to determine the pharmacokinetic parameters (Thermo Fisher, PA, USA). Peak plasma concentration (*C*_max_) and corresponding time (*T*_max_) were calculated from raw data. Area under the plasma concentration-time curve (AUC) was determined by linear/linear-log trapezoidal rule of non-compartmental model using data from time 0 and the last measurable time point (AUC_last_). All statistics were performed with Watson LIMS™ software. Plasma concentrations below the limit of quantitation (BLOQ) were treated as 0 for calculation.

### Aerosol Infection of Mice and Estimation of Bacterial Load

Female B6D2F1 mice were infected with Mtb CDC1551 using an aerosol-exposure system (CH Technologies Inc., Westwood, NJ, USA), as described in Ref. (11). The infection inoculum was adjusted to implant 100–150 bacterial colony-forming units (CFUs) in the lungs. To determine the actual number of bacilli inoculated, Mtb-infected mice (*n* = 6) were euthanized 3 h postinfection (*T* = 0), and serial dilutions of whole lung homogenates, prepared in sterile 1×PBS, were plated on Middlebrook 7H11 agar (Difco Laboratories Inc., Detroit, MI, USA); CFUs were counted following 4–6 weeks of incubation at 37°C in 5% CO_2._ Mtb-infected mice (*n* = 6) were euthanized at 14 days postinfection to determine the lung bacillary load (CFU) and pathology at the time of initiating treatment. At 14 days postinfection, Mtb-infected mice were allocated into four groups (*n* = 24 per group) and treated with CC-11050 (50 mg/kg), INH (50 mg/kg), or CC-11050 plus INH. The fourth group of Mtb-infected mice was left untreated as control. All drugs were dissolved in water and administered daily through oral gavage. At 28, 56, 84, and 112 days postinfection, mice from each group (*n* = 5–6 per group per time point) were euthanized, and portions of the lungs were used to prepare homogenates for CFU assay, stored in formalin for histologic analysis, or frozen immediately for RNA isolation. Serial dilutions of lung homogenates from these time points were plated on 7H10 agar supplemented with 0.2 or 1 μg/ml of INH to test for the emergence of INH-resistant Mtb; no CFUs were detected.

### Histologic Analysis

Formalin-fixed lung tissues of Mtb-infected mice were paraffin-embedded, cut into 5 μg sections, and stained with hematoxylin and eosin (H&E) or acid fast staining by Ziehl–Neelsen (ZN) method to visualize host cells and Mtb, respectively (IDEXX-RADIL Laboratories Inc., MO, USA) as described in Ref. (11). Stained sections were photographed in a Nikon Microphot-FX photomicrographic system and analyzed with NIS-Elements F3.0 software (Nikon Instruments Inc., NY, USA). Lung granuloma size was morphometrically measured using PathScan Enabler IV scanner (Meyer Instruments, Houston, TX, USA) and SigmaScan Pro software (Systat Software, Inc., CA, USA). Histopathologic analysis and morphometry were performed by an experienced pathologist blinded to the sample identity.

### RNA Isolation and Microarray Analysis

Total RNA was isolated from the lungs of uninfected or Mtb-infected mice with or without CC-11050 treatment (*n* = 4 per group) after 14 days of treatment using Trizol reagent (Invitrogen, CA, USA) as described in Ref. (11). One microgram of total lung RNA from each mouse was individually processed for cDNA synthesis using SuperScript II system (Agilent Technologies, CA, USA). The microarray experiment was performed using mouse genome ST 1.0 microarray kit (Affymetrix, Santa Clara, CA, USA), following a standard protocol^2^ for the data extraction and analysis. RMA-normalized, log_2_-transformed array data from individual mice from each group (*n* = 4 per group) were compared as follows (i) Mtb-infected versus uninfected and (ii) Mtb-infected CC-11050-treated versus untreated, using ANOVA in Partek Genomics Suite (Partek Inc., St. Louis, MO, USA). Significantly differentially expressed genes (SDEGs) were identified using 5% false discovery rate (FDR). The list of SDEGs was further analyzed using QIAGEN’s Ingenuity® Pathway Analysis (IPA®-QIAGEN, CA, USA) to determine significantly enriched (*P* < 0.05) gene networks and canonical pathways as described in Ref. (15). The microarray data has been submitted to GEO (Accession number GSE83188).

### Quantitative Real-time PCR

Total lung RNA (200 ng) was reverse-transcribed using Sprint RT kit (Clontech, CA, USA); 10 ng of cDNA were used for quantitative real-time PCR (qPCR), with Brilliant II SYBR master mix (Agilent Technologies, CA, USA) and gene-specific primers in a Stratagene Mx3005P instrument (Agilent Technologies, CA, USA). ROX was included in each reaction as an internal control. The qPCR assay was performed using four mouse samples in duplicate per group; the data were analyzed using MxPro Software (Agilent Technologies, CA, USA). Transcript levels of *Gapdh* were used as a reference against the test genes. Fold change in gene expression was calculated using the formula 2^−ΔΔCt^, where ΔCt is the difference in threshold cycle (Ct) between the target gene and *Gapdh*; and ΔΔCt is the ratio between untreated and CC-11050-treated samples. Primers were designed based on nucleotide sequences obtained from PrimerBank.^3^

### Statistical Analysis

Student’s *t*-test in GraphPad Prism (GraphPad Software, CA, USA) was used to analyze group-wise comparisons of the CFUs, qPCR, and morphometric data. *P* < 0.05 was considered statistically significant. ANOVA in Partek Genomic Suite version 6.6 was used for microarray gene expression analysis, and right-tailed Fisher’s exact test in ingenuity pathway analysis was used to determine the significance for pathway/network analysis as described in Ref. (15).

## Results

### Pharmacokinetics of CC-11050

A single oral administration of CC-11050 at each of three dose levels (5, 25, 50 mg/kg) demonstrated rapid absorption of the drug, with peak plasma concentrations reached by 1–2 h (*T*_max_) (Figure S1B in Supplementary Material). The average composite exposures of CC-11050, measured by area under the curve (AUC_last_) in the plasma of mice given 5, 25, and 50 mg/kg doses, were 1,080, 2,800, and 10,200 ng × h/ml, respectively. As compared to the response to a single oral dose of CC-11050 (50 mg/kg) alone, CC-11050 (50 mg/kg) given in combination with INH (50 mg/kg), resulted in a delayed *T*_max_ from 2 to 5 h (Table 1). In addition, a slight increase in the maximum plasma concentration of CC-11050 (*C*_max_) was noted in mice treated with CC-11050 plus INH, compared to CC-11050 alone (1,610 versus 1,410 ng/ml). Moreover, the AUC_last_ for mice treated with CC-11050 plus INH was slightly higher than for those treated with CC-11050 alone (13,900 versus 10,200 ng × h/ml, respectively).

**Table 1:** **Plasma levels of CC-11050 with or without INH in mice after a single oral dose**.

Sampling time (h)	Concentration (ng/ml)^a^	
	CC-11050 only	CC-11050 + INH
1	1,331.6 ± 136.97	905.35 ± 594.23
2	1,409.47 ± 140.85	1,309.39 ± 214.08
5	948.85 ± 128.7	1,609.18 ± 167.2
8	820.6 ± 265.98	1,271.73 ± 249.18
24	1.27 ± 1.1	4.96 ± 1.85
*T*_max_ (h)	2.0	5.0
*C*_max_ (ng/ml)	1,410	1,610
AUC_last_ (ng × h/ml)	10,200	13,900

*^a^Values shown are mean ± SD*.

### CC-11050 Treatment Reduces *Pde4* Expression in Mtb-Infected Mouse Lungs

**Figure 1:**
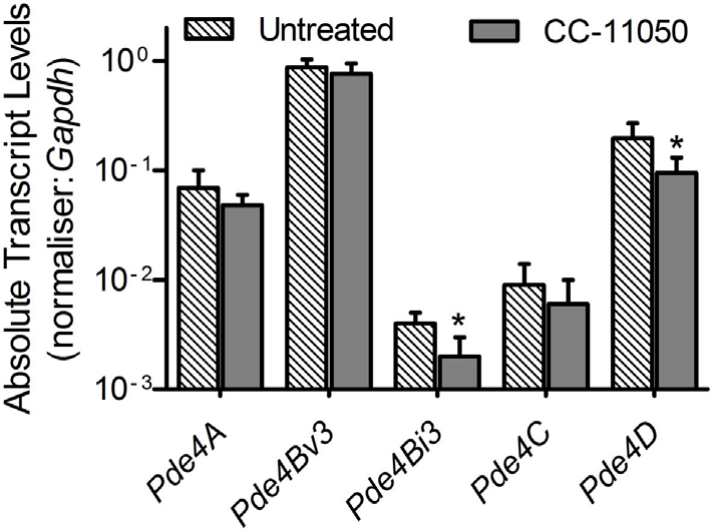
**Expression of host PDE4 genes in Mtb-infected untreated (hatched bars) or CC-11050-treated (solid bars) mouse lungs, relative to the uninfected controls**. Experiment was performed in duplicate with four biological replicates (*n* = 4 per group). *P = 0.0037 for *Pde4D* and *P* = 0.015 for *Pde4Bi3* between untreated and CC-1150-treated groups.

To test the impact of CC-11050 treatment on the expression pattern of *Pde4*, qPCR was performed using total RNA from the lungs of uninfected, Mtb-infected, and CC-11050-treated or untreated mice 14 days after initiation of treatment (i.e., 28 days postinfection) (Figure 1). Compared to the uninfected controls, Mtb infection induced the expression of all five genes tested in the lungs. Among these, CC-11050 treatment significantly reduced the expression level of *Pde4D* (*P* = 0.0037) and *Pde4Bi3* (Pde4B isoform 3; *P* = 0.015). No significant change in the expression of *Pde4A*, *Pde4Bv3* (Pde4B variant 3), and *Pde4C* was noted (Figure 1). Taken together, these results suggest that *Pde4D* and *Pde4Bi3* are potential targets of CC-11050, although this observation needs to be confirmed at the protein level.

### Genome-Wide Lung Transcriptome Analysis of Untreated and CC-11050-Treated Mtb-Infected Mice

**Figure 2:**
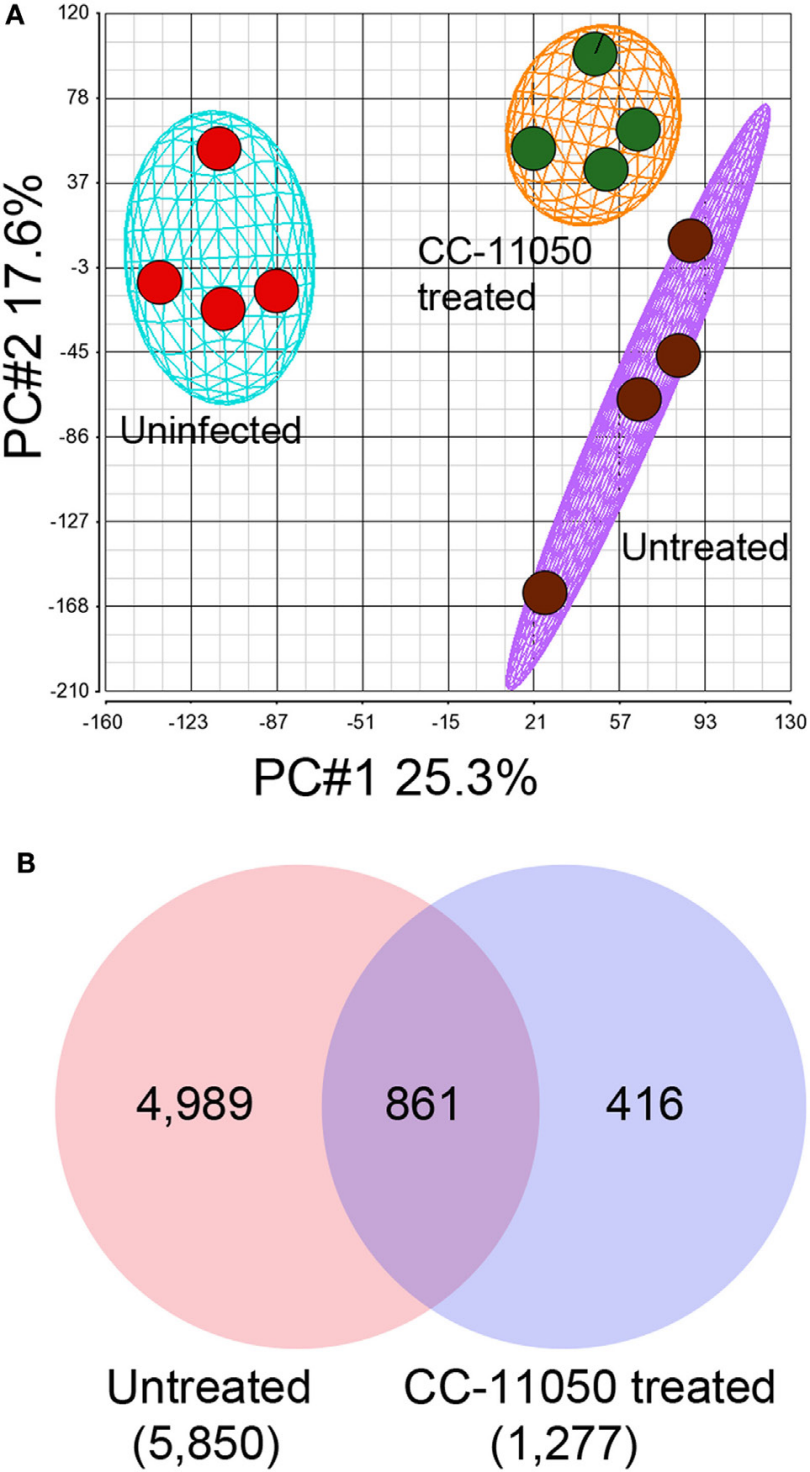
**Genome-wide lung transcriptome of Mtb-infected mice with or without CC-11050 treatment**. **(A)** Principal component analysis (PCA) map showing segregation of uninfected, Mtb-infected untreated, or CC-11050-treated samples (*n* = 4 per group). The eclipse/oval shape represents 2 × SD from the mean data. **(B)** Venn diagram showing unique and shared SDEGs between Mtb-infected untreated (red) and CC-11050-treated (blue) mouse lungs after 14 days of treatment. A false discovery rate (FDR) of 5% was applied to select the SDEGs. Microarray data were generated with four biological replicates per each group. Numbers in parenthesis are the total SDEGs.

A microarray-based global gene expression profiling was used to determine the effect of CC-11050 treatment on Mtb-infected mouse lungs at 14 days of treatment. Principal component analysis (PCA) of the normalized data shows distinct clustering of the uninfected and Mtb-infected with or without CC-11050 treatment groups (Figure 2A). The PCA map also shows that the median expression level (±2SD) data within individual groups segregate closely and far from the other groups. This observation shows a high level of reproducibility of variables (state of infection and treatment) within and between the data obtained from different groups. With an FDR of 5% applied to the array data, we found 5,850 SDEGs in the lungs of Mtb-infected mice, relative to uninfected controls. In the Mtb-infected CC-11050-treated mice, 1,277 SDEGs were observed, relative to the infected untreated animals. Of these, 861 SDEGs were shared between the treated and untreated Mtb-infected groups (Figure 2B). Among the total genes, the expression of 875 SDEGs (685 upregulated and 190 downregulated) in the untreated and 47 SDEGs (29 upregulated and 18 downregulated) in the CC-11050-treated group were affected by more than 1.5-folds. Taken together, the microarray data suggest that Mtb infection significantly affects the expression of thousands of host genes in the lungs. Of these, the expression of a subset of these (14.7%) was significantly affected in the CC-11050-treated infected mice. Selected SDEGs from the microarray, including *Cyp10*, *Ly6a*, *Trdn*, *Irg1*, *Cxcl10*, *Ifng*, and *Tnfa*, were confirmed by qPCR. As shown in Figure S2 in Supplementary Material, the expression pattern of these genes was consistent between microarray and qPCR.

### Gene Ontology Analysis of SDEGs

To determine the biological functions affected by the differential host gene expression in the Mtb-infected untreated and CC-11050-treated mouse lungs, we analyzed respective sets of SDEGs using IPA (Table 2). The gene ontology (GO) analysis revealed that host cell processes associated with infectious disease and inflammatory response were among the most significantly affected biological functions in the Mtb-infected untreated group. This is consistent with the progressive pathology seen in the mouse lungs (see below). In the infected CC-11050-treated animals, biological functions associated with infectious disease, gene expression, and host cell assembly and organization were among the most significantly affected. As shown in Table 2, the biological functions affected in the untreated group were of a higher level of significance (10^−34^–10^−07^), compared to a moderate level of significance for the CC-11050-treated group (10^−06^–10^−02^). Taken together, the GO analysis suggests distinct changes in the biological functions affected in the mouse lungs by Mtb infection with or without CC-11050 treatment.

**Table 2:** **Gene ontology analysis of significantly differentially expressed genes**.

Biological function	*P* value
**Mtb-infected untreated versus uninfected**
Infectious disease	8.36E−34–4.77E−08
Immunological disease	4.51E−31–1.36E−07
Inflammatory response	7.45E−31–2.26E−08
Cellular function and maintenance	8.82E−48–1.08E−07
Cellular growth and proliferation	8.54E−34–1.12E−07
Cell death and survival	1.03E−33–6.24E−08
Cellular movement	5.24E−33–1.27E−07
Cellular development	2.52E−32–1.12E−07
**CC-11050**-**treated versus untreated**
Infectious disease	8.53E−05–1.64E−02
Gene expression	1.47E−06–1.80E−02
Cellular assembly and organization	2.65E−05–2.38E−02
Cell morphology	6.44E−05–2.38E−02
Cellular growth and proliferation	8.73E−05–2.29E−02
Cellular function and maintenance	1.02E−04–2.38E−02

### Selected Immune Network/Pathway Analysis of SDEGs

Based on the GO results, we extended our analysis to explore the TNF-α interaction, lung inflammatory response, Il-17, and interferon (Ifn) signaling networks/pathways. These networks/pathways play key roles in the host immune/inflammatory response to Mtb infection and are likely to be affected by the anti-inflammatory activities of PDE4 inhibitors (16). The expression pattern of selected SDEGs in these networks/pathways was validated by qPCR (Figure S2 in Supplementary Material).

#### TNF-α Interaction Network

**Figure 3:**
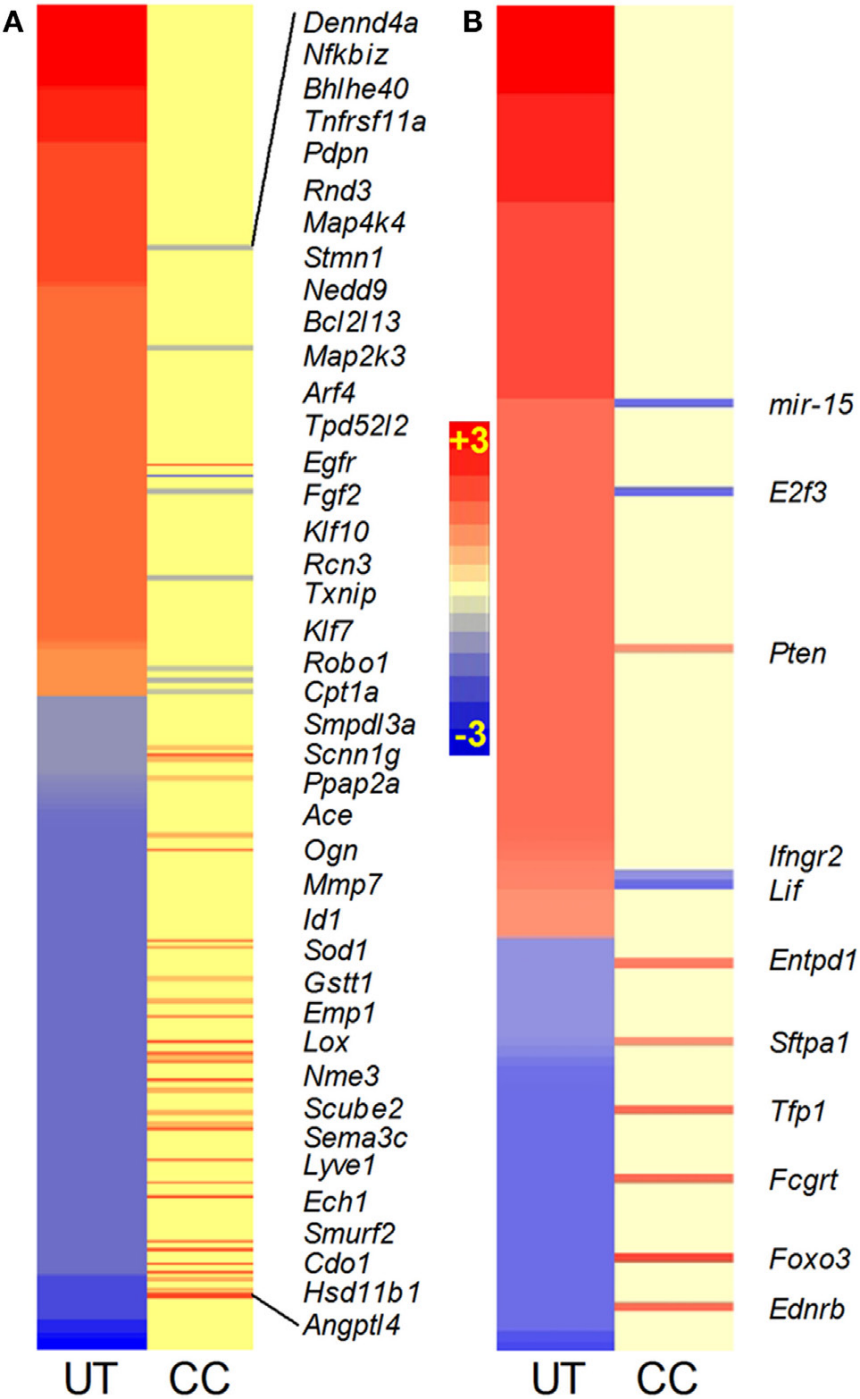
**Intensity plot of TNF-α (A) and lung inflammatory response (B) network gene expression in Mtb-infected mouse lungs with or without CC-11050 treatment**. UT, untreated; CC, treated with CC-11050. Only genes expressed in CC are shown in italicized gene symbols. Red color shows upregulation and blue color shows downregulation of gene expression. Scale bar ranges from +3 (red) to −3 (blue).

Among the SDEGs in the Mtb-infected untreated mouse lungs, 479 were associated with the TNF-α interaction network. Of these, 246 genes were upregulated, including 51 genes induced by more than twofold, while 234 genes were downregulated (Figure 3A; Table S1 in Supplementary Material). The expression of TNF-α-mediated inflammatory molecules, including *Cxcl9*, *Saa2*, *Cxcl10*, and *Irg1* (Table S1 in Supplementary Material), was upregulated by more than 10-fold in the Mtb-infected untreated animals. Compared to this, 33 SDEGs were upregulated, and 8 SDEGs were downregulated in the lungs of CC-11050-treated infected animals (Figure 3A; Table S1 in Supplementary Material). In these animals, none of the SDEGs was altered by more than a twofold change in expression. Thus, CC-11050 treatment significantly dampened the expression of many SDEGs in the TNF-α network that were upregulated in response to Mtb infection in the mouse lungs.

#### Lung Inflammation Network

TNF-α is one of the key proinflammatory molecules involved in driving the host inflammatory response during progressive TB (16). Therefore, we investigated the expression profile of the lung inflammation network in the Mtb-infected untreated and CC-11050-treated mouse lungs (Figure 3B; Table S1 in Supplementary Material). To avoid repetition, SDEGs analyzed in the TNF-α interaction network were excluded from the list of genes included in the lung inflammation network. A subset of 137 out of 5,780 SDEGs in the infected untreated mouse lungs were associated with the inflammatory response. Of these, 95 genes were upregulated, including *I11*β**, *Il6*, *Mmp14*, *Ccr5*, *Nos2*, *Mcp1*, *Stat1*, and *Cxcr3*, while 42 genes were downregulated. In comparison, nine genes were upregulated, and four were downregulated in the infected CC-11050-treated animals (Figure 3B; Table S1 in Supplementary Material). Thus, consistent with the expression pattern of the TNF-α network, many of the SDEGs in the inflammatory response were upregulated by Mtb infection. These results suggest that CC-11050 dampened the expression of key inflammatory response molecules triggered by Mtb infection in the treated mouse lungs.

#### Il-17 Interaction Network

**Figure 4:**
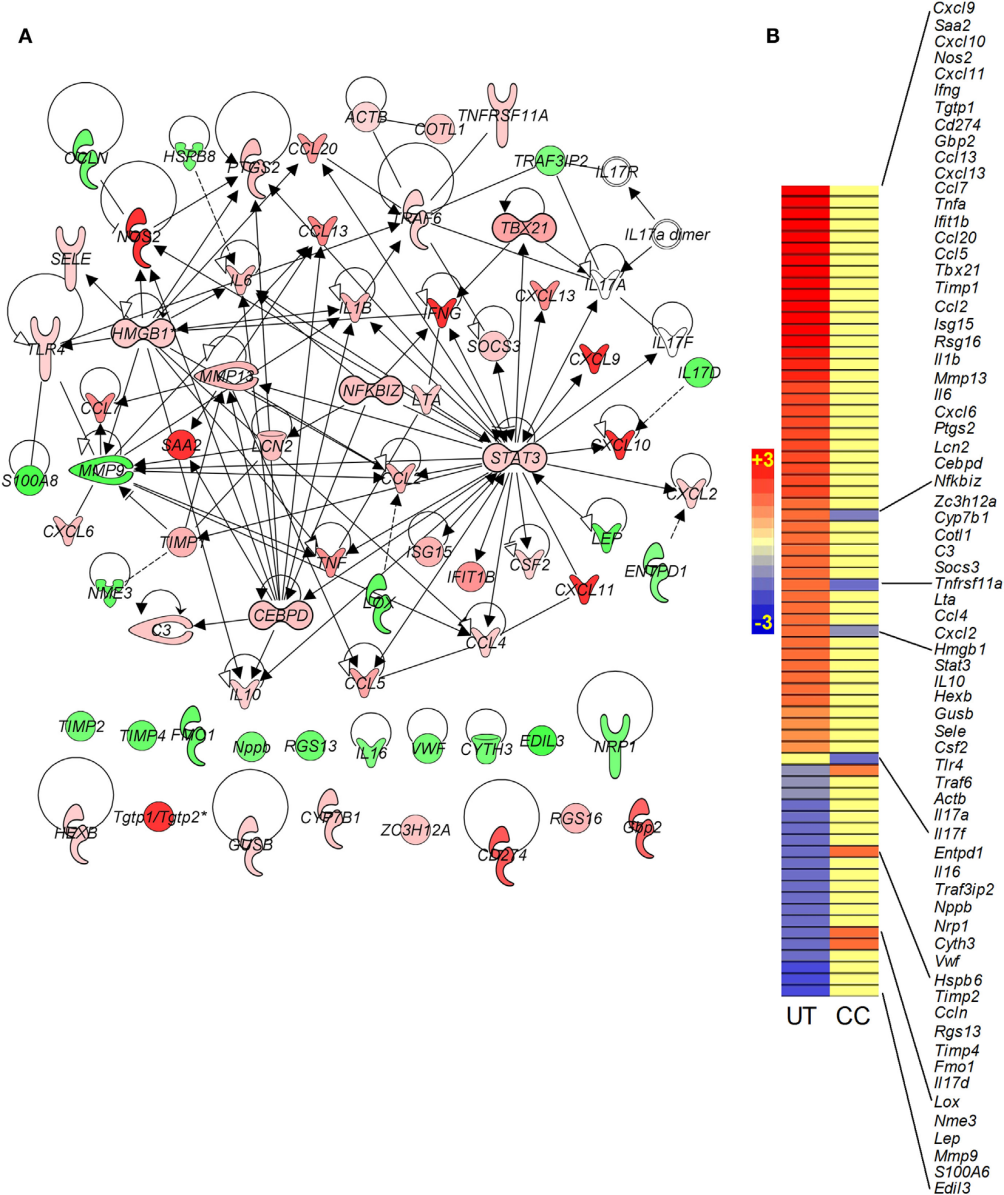
**Gene interaction network and intensity plot of SDEGs associated with Il-17 in Mtb-infected mouse lungs with or without CC-11050 treatment**. **(A)** Interaction of member genes involved in Il-17 expression/regulation. Solid lines represent direct connection, and broken lines indicate indirect link. Red color indicates upregulation, and green color depicts downregulation. **(B)** Intensity plot of SDEGs associated with Il-17 network. UT, untreated; CC, treated with CC-11050. Red color shows upregulation, and blue color denotes downregulation of gene expression. Scale bar ranges from +3 (red) to −3 (blue).

Recently, Il-17 has been implicated as a crucial cytokine in the host immune/inflammatory response to Mtb infection (17). We probed the SDEGs from Mtb-infected untreated and CC-11050-treated mouse lungs to examine the differential expression of genes associated with *Il17*. Of the total SDEGs, 30 genes were found to be associated with the Il-17 interaction network. Of these, Mtb infection resulted in upregulation of 20 genes, including *Nos2*, *Ifn*γ, *Ccl13*, *Tnf*α, and *Ccl5*, which were induced by more than twofold, and downregulation of 9 genes (Figures 4A,B). In the infected untreated animals, *Il17D* was downregulated, while no significant change was noted in *Il17A* and *Il17F* expression. In contrast, only four genes (*HmgB1*, *Il17F*, *Entpd1*, and *HspB8*) were significantly differentially expressed in the lungs of infected CC-11050-treated mice; all of which were affected by less than twofold (Figure 4B). In summary, while 97% of genes in the Il-17 interaction network were significantly affected by Mtb infection, the expression of 87% of these genes was dampened by CC-11050, suggesting downregulation of this network in the infected treated mouse lungs.

#### Canonical IFN Signaling Pathway

**Figure 5:**
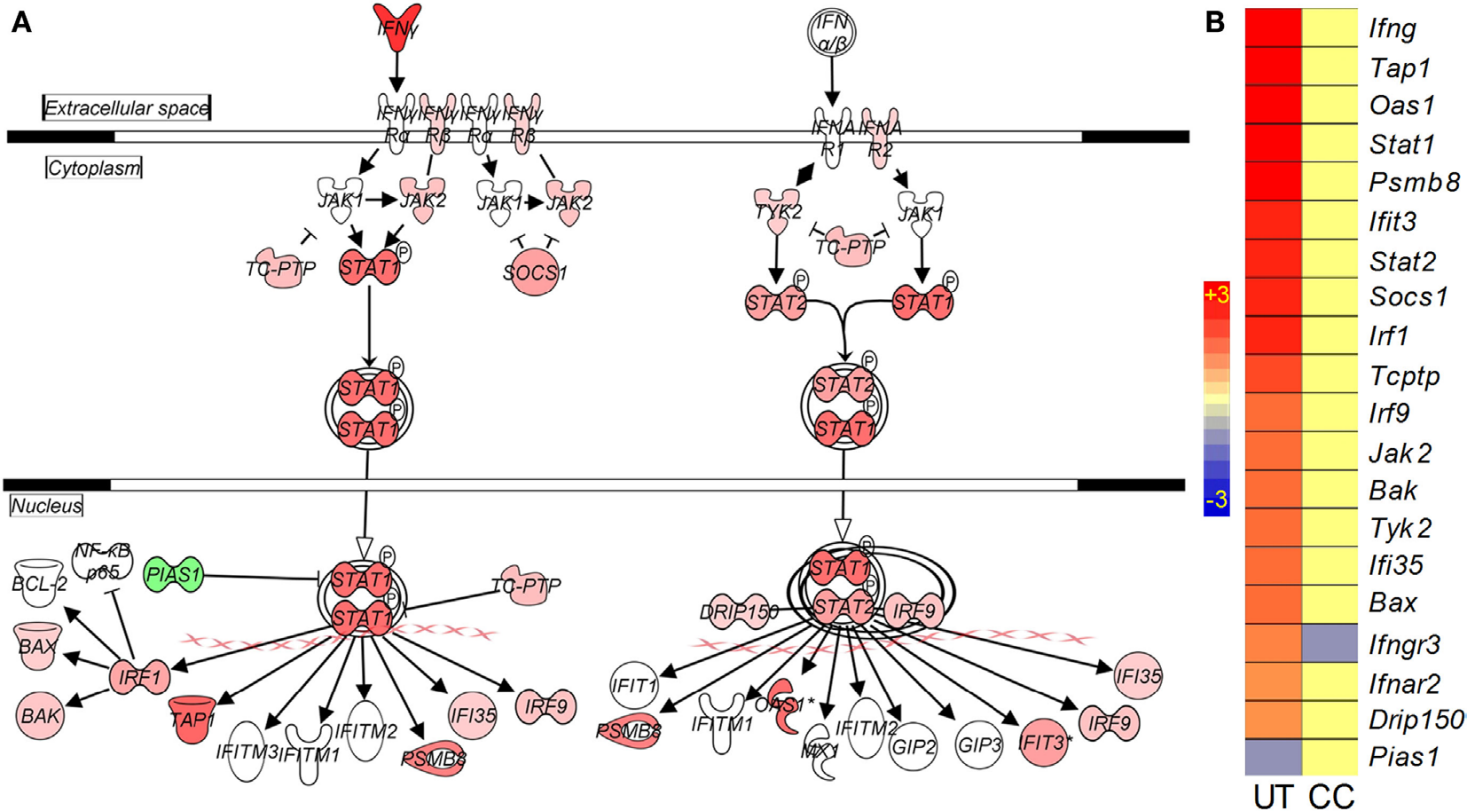
**Interaction and expression pattern of SDEGs involved in canonical interferon signaling pathway in Mtb-infected mouse lungs with or without CC-11050 treatment**. **(A)** Pathway map showing interaction of member genes involved in Ifn signaling pathway. Red color indicates upregulation, and green color indicates downregulation. **(B)** Intensity plot of SDEGs associated with Ifn signaling. UT, untreated; CC, treated with CC-11050. Red color shows upregulation, and blue color shows downregulation of gene expression. Scale bar ranges from +3 (red) to −3 (blue).

Interferon signaling is a hallmark of the onset of the adaptive immune response to Mtb infection in mouse lungs (16). As expected, many genes in this pathway, including *Ifn*γ, *Stat1*, *Stat2*, *Irf1*, *Irf9*, *Ifi35*, and *Ifit3*, were upregulated in the mouse lungs by Mtb infection (Figures 5A,B). However, in the infected CC-11050-treated animals, with the exception of *Ifngr3* that was downregulated, the expression of all other genes in this pathway remained at levels similar to uninfected controls (Figure 5B; Table S1 in Supplementary Material).

### CC-11050 Plus INH Treatment Reduces Bacillary Load in Mtb-Infected Mouse Lungs

**Figure 6:**
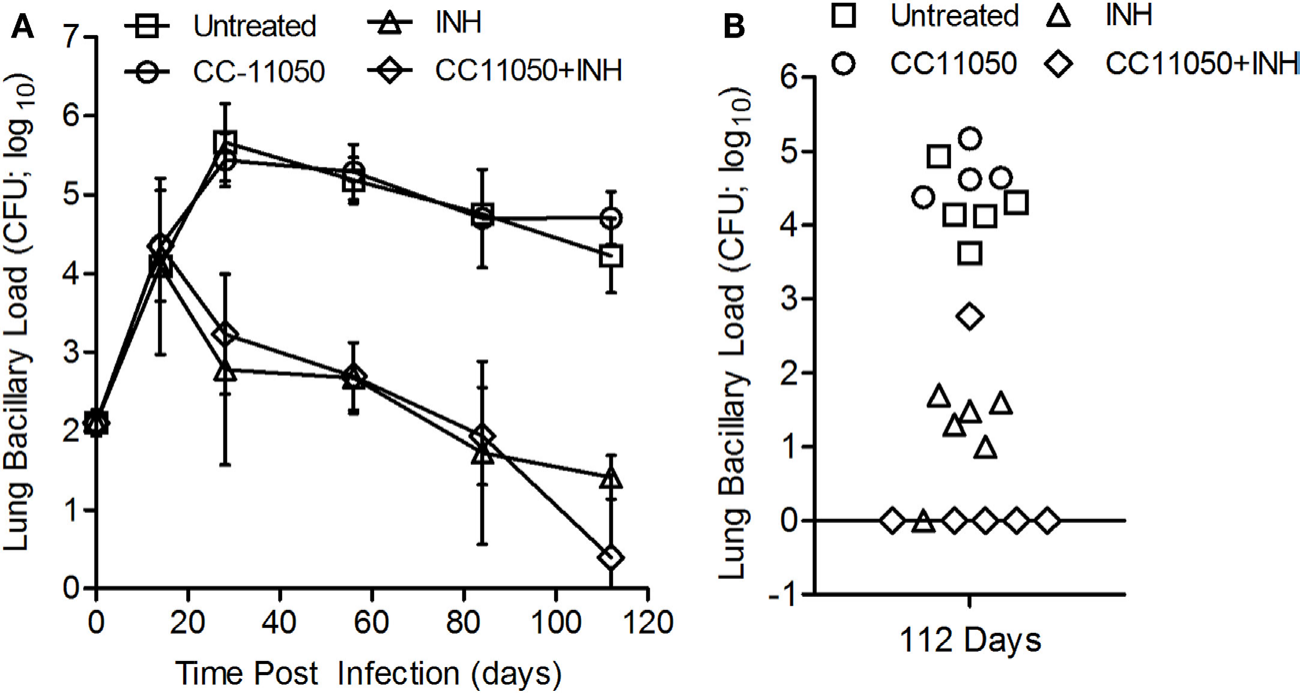
**Bacillary load in Mtb-infected mouse lungs with or without CC-11050 and/or INH treatment**. **(A)** Bacterial CFUs at various time points in different treatment groups. Treatment was started at 14 days postinfection. *P = 0.07 between INH (triangle) and INH plus CC-11050-treated groups (diamond) (*n* = 6 per group per time point). **(B)** Individual data points at 112 days. One animal out of six from each of INH or INH plus CC-11050-treated groups had unusual CFU pattern that can be an outlier or due to technical error. Excluding these two samples from the analysis led to a significant difference in lung CFUs at 112 days postinfection between the two treatment groups (*P* = 0.02; *n* = 5 per group).

To determine the impact of CC-11050 on treatment of Mtb-infected mice with INH, we enumerated the lung bacillary load in all treatment groups over the duration of infection (Figure 6). The inoculum at time 0 (3 h postinfection) was about 2 log_10_ CFU. At the onset of treatment (14 days postinfection), the bacillary load had reached 4.2 log_10_ CFU. In mice left untreated or treated with CC-11050 alone, the bacillary load increased to about 5.5 log_10_ by 28 days and remained at persistently high levels until 112 days postinfection in both groups (Figure 6A). No significant difference in the number of lung CFUs was noted between untreated and CC-11050-treated mice at any of the time points tested, indicating that CC-11050 does not have anti-Mtb activity and does not cause generalized immune suppression in the animals. In the mice treated with INH or INH plus CC-11050, we observed a gradual reduction in the lung CFUs from 28 until 84 days postinfection, after which the INH alone group showed no significant decline to 112 days. In contrast, the CFUs continued to decline in the CC-11050 plus INH treatment group, reaching undetectable levels in the lungs of five out of six animals (*P* = 0.02) by 112 days postinfection (Figures 6A,B). Moreover, lung homogenates from infected mice treated with CC-11050 plus INH yielded no detectable CFUs when plated on media containing INH, indicating that PDE4 inhibition was not associated with the emergence of resistant organisms.

### CC-11050 Plus INH Treatment Reduces Lung Pathology in Mtb-Infected Mice

**Figure 7:**
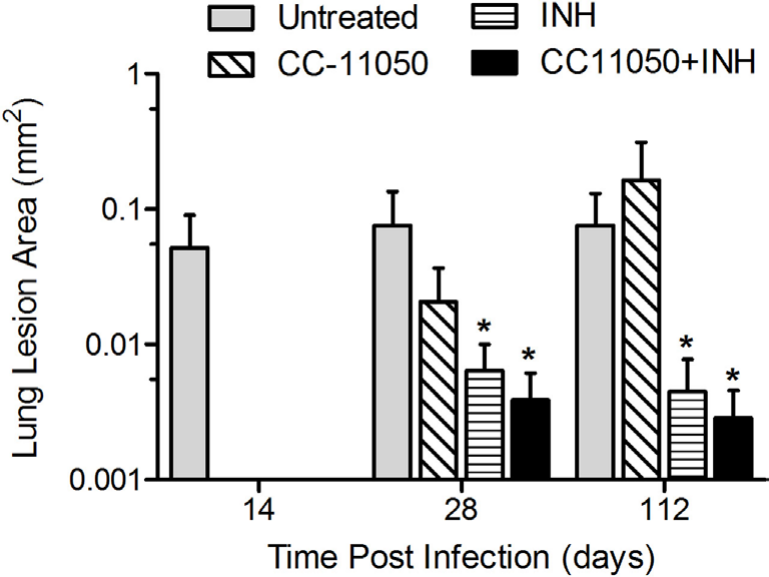
**Morphometric measurements of lung granulomas in Mtb-infected mice treated with CC-11050 (hatched bars), INH (horizontal bars), both (solid bars), or none (gray bars)**. *P = 0.0302 between INH and no treatment; *P* = 0.0259 between INH plus CC-11050 and no treatment at 28 days postinfection. *P = 0.008 and 0.0371, respectively, for INH and INH + CC-11050, compared to no treatment group (*n* = 4–6 per group per time point).

A morphometric analysis was carried out to compare the extent of pathology in the lungs of Mtb-infected mice in the different treatment groups (Figure 7). In the Mtb-infected untreated mice, the size (area) of granulomas at 28 days postinfection reached about 0.08 ± 0.059 mm^2^. At this time, significant differences in the lesion area were already noted between the untreated mice and those treated with INH alone (*P* = 0.0302) or with INH plus CC-11050 (*P* = 0.0259). By 112 days postinfection, a significant reduction in granuloma size was noted in the lungs of mice treated with INH, with or without adjunctive CC-11050, compared to the untreated group (*P* = 0.008 and 0.0371, respectively, for INH and INH plus CC-11050). Infected mice treated with INH plus CC-11050 had smaller lesions, compared to those treated with INH alone, although the difference was not statistically significant (Figure 7).

**Figure 8:**
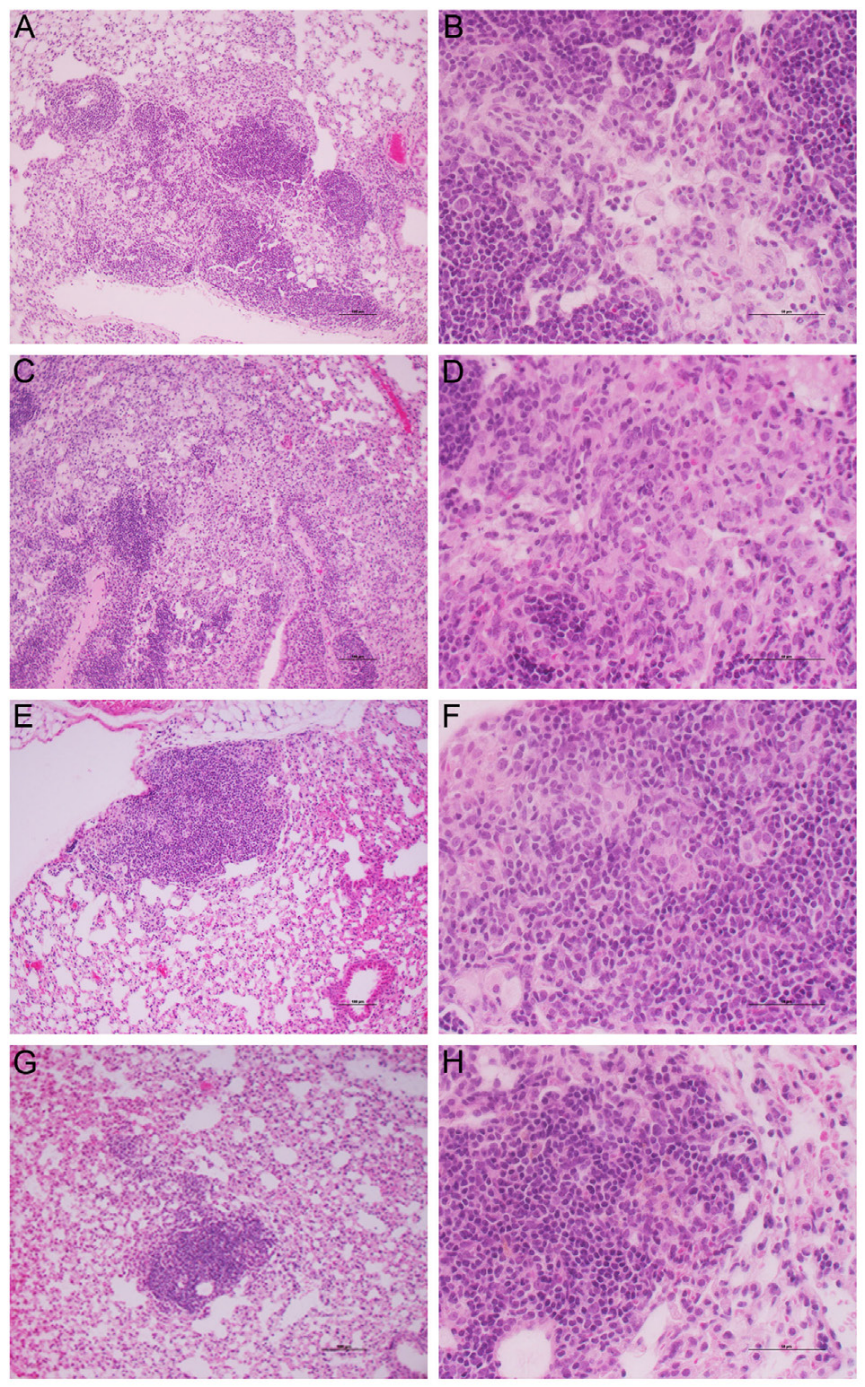
**Histology of Mtb-infected mouse lungs at 112 days postinfection**. Representative image of H&E stained mouse lung sections at 10× **(A,C,E,G)** and 40× **(B,D,F,H)** magnifications. **(A,B)** untreated; **(C,D)** CC-11050-treated; **(E,F)** INH-treated; and **(G,H)** INH plus CC-11050-treated. **(A,C,E,G)** are photographed at 10×, and **(B,D,F,H)** are photographed at 40× magnification. Note the presence of multiple, coalescent, and larger granulomas in the untreated and CC-11050-treated mouse lungs **(A–D)**; in contrast, the INH-treated mouse had a smaller **(E,F)**, while the INH plus CC-11050-treated animals had the smallest lung lesion **(G,H)**.

Histologic examination of the lungs also revealed differences in pathology between the treatment groups. At 28 days postinfection (14 days of treatment), the lungs of untreated mice showed diffuse granulomas (0.5–1 mm diameter), comprising aggregates of evenly dispersed macrophages and lymphocytes with few polymorphonuclear cells (Figures S3A,B in Supplementary Material). At this time, treatment with CC-11050 alone had no significant impact on the size and structure of the lesions, confirming that CC-11050 was not immune-suppressive (Figures S3C,D in Supplementary Material). In mice treated with INH alone (Figures S3E,F in Supplementary Material) or INH plus CC-11050 (Figures S3G,H in Supplementary Material), the lesions were somewhat smaller than those in the untreated or CC-11050-treated groups with much smaller areas of aggregated lymphoid cells. By 112 days postinfection, the relative extent of disease among the Mtb-infected untreated groups was similar to those observed at 28 days, although the granulomas showed a more structured cellular organization (Figure 8). In the lungs of untreated mice, the granulomas had condensed somewhat from the earlier time point and were beginning to coalesce (Figures 8A,B). These lesions contained small aggregates of macrophages with some scattered lymphocytes, surrounded by regions of more concentrated lymphoid cells. Treatment of the infected mice with CC-11050 alone did not result in significant differences in the immune cell composition of lung lesions from those seen in the untreated animals (Figures 8C,D). In contrast, the granulomas in the lungs of mice treated with INH alone were smaller and comprised mainly of lymphocytes surrounding small patches of macrophages at the center (Figures 8E,F). Treatment with CC-11050 plus INH resulted in fewer and more condensed lung lesions, compared to the other groups. These lesions were composed of densely packed lymphocytes with few interspersed macrophages (Figures 8G,H).

## Discussion

Host-directed therapy shows promise as an adjunct to conventional antibiotic treatment for TB to shorten the duration of chemotherapy and improve outcome (8, 18). The study described here extends our previous work demonstrating that selective immune modulation with a small molecule PDE4i, as an adjunct to the anti-TB drug INH, can reduce pathology and enhance antimicrobial activity in the mouse model of pulmonary TB (11–13).

In this study, we compared the impact of CC-11050 on TB treatment with our previous findings for CC-3052. Our results confirm and expand our previous observations and provide some information on the lung-specific PDE4 isoforms targeted by CC-11050. Previous reports have indicated that not all small molecule PDE4i drugs have the same capacity to improve TB chemotherapy (19–22). While rolipram and cilomilast treatment of Mtb-infected mice showed no impact on antibiotic-mediated bacillary clearance in the mouse pulmonary TB model (19), co-treatment of mice with roflumilast plus INH resulted in significantly reduced CFUs in the lungs, compared to INH alone (20), similar to our findings with CC-3052 and CC-11050. The variation in action of these molecules may reflect diversity in the enzyme specificities of the inhibitors. The family of PDE4 enzymes has 4 known subtypes (PDE4A–D) and at least 15 isotypes, which are differentially expressed in various tissue and cell types and differentially targeted by specific inhibitors (23). For example, the adverse gastrointestinal side effects, which limit pharmacologic use of rolipram, have been associated with specific splice-variant isoforms of *Pde4D* (21), while roflumilast appears to exert its anti-inflammatory effects mainly through PDE4B (22). In the present study, treatment with CC-11050 alone led to reduced transcript levels of *Pde4D* and *Pde4Bi3* in the lungs, suggesting that these isoforms may be specifically targeted. However, our analysis did not distinguish between *Pde4D* splice-variants, and the effect of CC-11050 on these targets remains to be confirmed at the protein level.

The pharmacokinetic experiment revealed more rapid and greater absorption of CC-11050 in the mice than we observed in rabbits at the same dose of 50 mg/kg (*T*_max_ and *C*_max_ of 1,410 ng/ml and 2 h versus 163 ng/ml and 4 h, respectively) (14). These findings may reflect differences between rabbits and mice in gastrointestinal absorption, metabolism, tissue distribution, and/or excretion of the PDE4i. Coadministration of the PDE4i with INH to the mice resulted in a delay of the CC-11050 *T*_max_ to 5 h, and a moderate increase in plasma *C*_max_ and AUC_last_. These observations are likely explained by the well-documented inhibitory effects of INH on drug metabolism, mainly through its inhibition of several cytochrome *P* isoforms in the liver (24). In contrast, our previous study in rabbits showed no effects of INH on the pharmacokinetics of CC-11050 (14). Moreover, the PDE4i caused no significant changes in plasma levels of INH in co-treated rabbits. This observation may be relevant to the potential emergence of resistant bacilli when patients are treated with anti-TB drugs and adjunctive HDT. Of note, no INH-resistant bacilli have been recovered from any co-treated rabbits or mice in our investigations of PDE4i (11). Despite the differences in pharmacokinetics between the two animal models, the effects of CC-11050 on host gene expression and INH-mediated Mtb clearance were similar, supporting the likelihood for similar responses in humans. Taken together, the results of our studies in both the rabbit and mouse models provide a rationale for phase I clinical trials to establish appropriate dosing and determine the potential for drug–drug interactions between CC-11050 and antimicrobials in humans.

The GO analysis revealed that host cell processes associated with infectious disease, inflammatory response, and cellular function were among the most significantly affected biological functions in the untreated Mtb-infected mice. We observed a similar transcriptome profile in the lungs of Mtb-infected rabbits, in addition to significant changes in genes involved with lipid metabolism and small molecule transport (14). These additional biological functions may reflect differences between the physiological response of mice and rabbits to Mtb infection, particularly with regard to the formation of differentiated granulomas, which are seen in rabbits but not in mice (25). Mtb-infected mice develop lesions within the lungs, composed of immune cell aggregates with minimal structure and no hypoxia, which enlarge and coalesce over time. In contrast, rabbits show a progressive granulomatous response to Mtb infection, with highly structured hypoxic lesions surrounded by a fibrotic capsule. These lesions eventually liquefy and form open cavities, similar to the response seen in humans with pulmonary TB (25). Despite these differences, both models showed reduced signs of pathology in response to CC-11050 adjunctive treatment (14). This was demonstrated in mice by the reduced size in lung lesions of CC-11050 plus INH-treated animals. Additionally, MMP14 and several genes in the fibrosis network, which were upregulated by infection, were downregulated to basal (uninfected) levels in response to CC-11050 treatment alone. Thus, despite the lack of visible fibrosis in the lungs of Mtb-infected mice, these animals displayed a gene expression profile in response to PDE4 inhibition that was consistent with the pathologic changes seen in Mtb-infected rabbits receiving the same treatment.

Although the rabbit model of pulmonary TB effectively mimics the pathology of human disease, the rabbit genome has only been completed to 7× coverage, with roughly 80% of the genes annotated on the basis of predictions with limited functional validation (Broad Institute). For this reason, the mouse model provided a useful validation for our transcriptomic studies, particularly with regard to the early immune response to Mtb infection, which has been well studied in this animal. Genome-wide lung transcriptome analysis revealed dampened inflammatory response-associated network genes in CC-11050-treated mice. Specifically, several genes of the TNF-α, lung inflammation, Il-17, and Ifn signaling pathways/networks were upregulated in mouse lungs by Mtb infection; expression of most of these genes was significantly reduced by CC-11050 treatment.

Treatment of mice with CC-11050 plus INH led to a significant improvement in antibiotic-mediated bacillary clearance from the lungs, compared to INH alone. Similar to our previous results for CC-3052 in Mtb-infected mice, CC-11050 did not significantly affect the kinetics of bacillary clearance at early time points, whereas at later time points, when INH alone showed a plateau in Mtb killing, the PDE4i co-treated animals continued to show a decline in CFUs, reaching undetectable levels in 5/6 animals by 112 days postinfection. This alteration of the biphasic kill curve seen with INH alone is consistent with our hypothesis that modulation of the host innate immune response can lead to a reduction in the population of bacilli shifting into a physiologic state in which they are refractory to antibiotic killing (26). The association between host environmental pressure and antibiotic tolerance of Mtb was demonstrated in a recent study by Liu et al., where activation of Mtb-infected macrophages *in vitro* rendered the bacilli tolerant to four first-line TB drugs, each of which was able to kill Mtb in resting macrophages (27). Transcriptional analysis of the intracellular bacilli revealed a common set of genes that were differentially expressed upon exposure to each of the four antibiotics. Moreover, the perturbations in the bacillary transcriptome caused by anti-TB drugs were strikingly similar to the profile of differentially expressed Mtb genes observed inside activated, compared to resting, macrophages (27). Thus, observations from the Liu et al., study suggest that the environmental pressure on Mtb within activated macrophages induces a stress response that renders the organisms tolerant to anti-TB drugs.

The safety of small molecule PDE4i as anti-inflammatory agents is an important consideration in the selection of these drugs for use in humans. Both mice and rabbits treated with CC-11050 or CC-3052 alone retained their ability to control Mtb growth as the infection progressed, indicating that adaptive immunity was not adversely affected. In contrast, uncontrolled bacillary replication in the lungs was a characteristic of generalized immune suppression, induced in mice by treatment with anti-TNF antibody or in rabbits treated with the soluble TNF receptor etanercept (11, 28).

Thus far, we have evaluated PDE4i in the context of treatment of mice with a single anti-TB drug, INH. Because of the steep killing curve observed when infected mice are initially exposed to antibiotic, it may be difficult to see the additive effect of a second antibiotic. In contrast, the killing curve in rabbits, where the organisms are within fully differentiated granulomas, is much slower, providing an opportunity to evaluate the impact of CC-11050 on a multidrug regimen (14, 29). Consequently, we are evaluating the efficacy of CC-11050, provided as an adjunct, in combination with two first-line anti-TB drugs, INH plus RIF, in the rabbit model of pulmonary Mtb infection. Our preliminary results indicate that, after 8 weeks of treatment (starting at 4 weeks postinfection), the combination of RIF plus INH shows enhanced bacillary killing compared to INH alone. Here, again, co-treatment of rabbits with CC-11050, together with INH and RIF, shows reduced CFUs in the lungs compared to INH and RIF alone. This study is currently in progress, and the results will be reported in a future publication.

In conclusion, the results of our present and published studies, conducted in both the mouse and rabbit models of pulmonary TB using two alternative inhibitors, provide proof-of-concept that adjunctive PDE4i significantly improves antibiotic-mediated bacterial killing and reduces lung pathology and is a promising candidate drug for HDT to improve treatment of humans with pulmonary TB.

## Author Contributions

SS, M-SK, JZ, and GK designed the study. SS and M-SK performed the experiments. SS, M-SK, LT, VK, JZ, DF, and GK analyzed and interpreted the data. VK and JZ supplied additional reagents for this study. SS, M-SK, LT, DF, and GK wrote the manuscript. All authors have read, reviewed, and edited the manuscript and agreed for submission to this journal.

## Conflict of Interest Statement

VK and JZ are employees of Celgene Corporation. GK is a member of the board of Celgene Corporation. All other authors: none to declare.

## Footnotes

^1^http://www.who.int/tb/Global_TB_Facts.pdf?ua=1

^2^http://www.cag.icph.org/index.htm

^3^https://pga.mgh.harvard.edu/primerbank/

## Supplementary Material

Figure S1 **Structure of CC-11050 (A) and plasma levels of CC-11050 at various time points up to 24 h in uninfected mouse treated with various concentrations (B)**.

Figure S2 **Validation of microarray data by qPCR**. Randomly selected mouse genes from microarray data at 28 days postinfection was evaluated by qPCR. Values shown are expression levels in CC-11050-treated mouse lungs, compared to the untreated. Level of Gapdh expression was used to normalize the data. The experiment was repeated in duplicate with four samples per group. ****P*** < 0.05; ***P* < 0.001.

Figure S3 **Histology of mouse lung at 28 days postinfection**. Representative images of mouse lung sections at 28 days postinfection (14 days posttreatment), stained with H & E, showing the architecture of granulomas with diffuse accumulation of inflammatory/immune cells. **(A,B)** untreated; **(C,D)** CC-11050-treated; **(E,F)** INH-treated; **(G,H)** INH plus CC-11050-treated. **(A,C,E,G)** are photographed at 10×, and **(B,D,F,H)** are photographed at 40× magnification. Note that the granulomas in INH or INH plus CC-11050-treated mouse lungs are smaller than the untreated or those treated with CC-11050 alone.

Table S1 **List of SDEGs associated with TNF-α, lung inflammation, Il-17, and Ifn signaling networks/pathways**.
