# Supplementary figures and images for: Pharmacologic Inhibition of Host Phosphodiesterase-4 Improves Isoniazid-Mediated Clearance of Mycobacterium tuberculosis
Source: Front Immunol. 2016 Jun 17;7:238. doi: 10.3389/fimmu.2016.00238 (PMC4911353; doi:10.3389/fimmu.2016.00238)

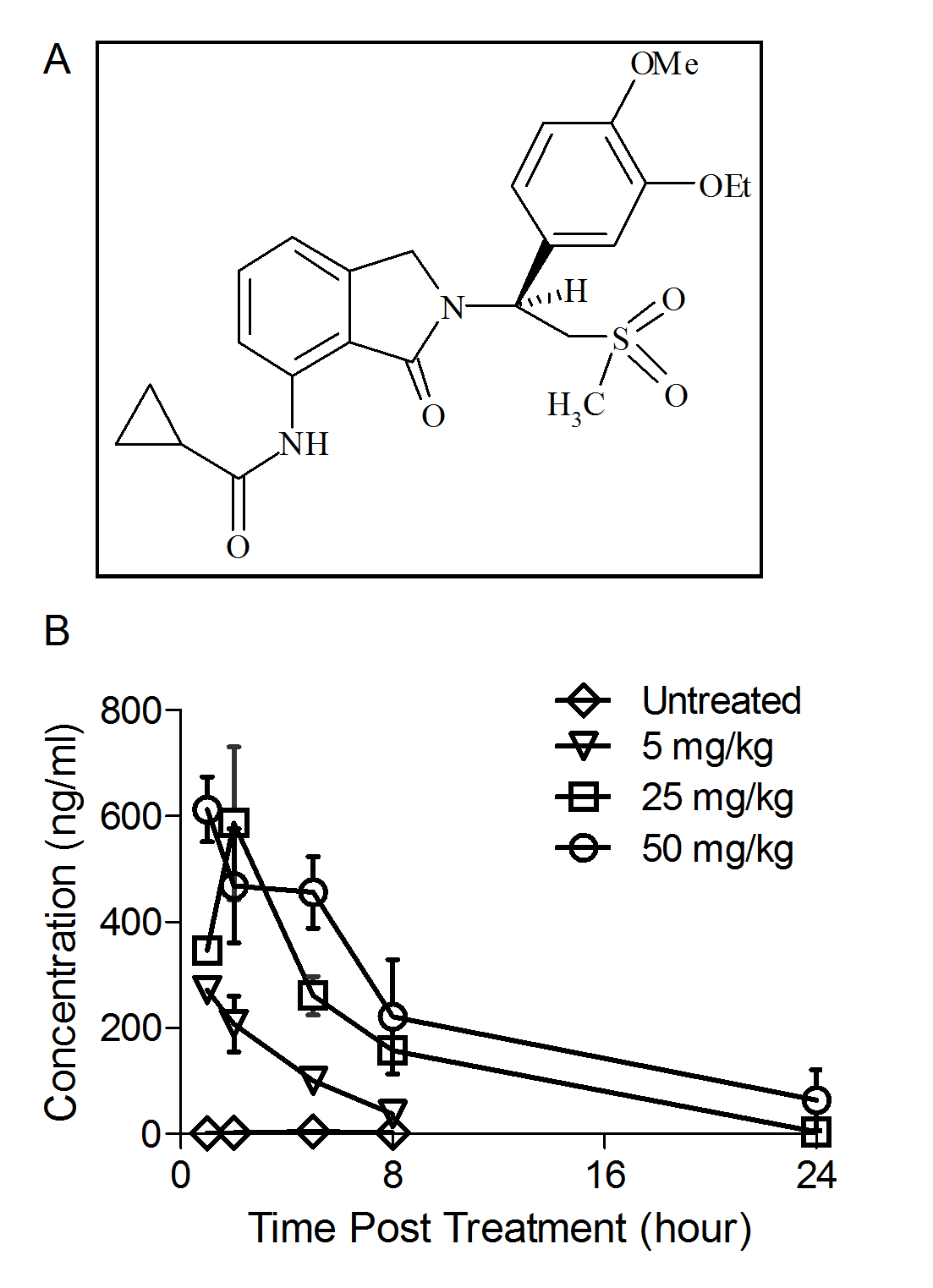

Supplement: Figure S1 — Structure of CC-11050 (A) and plasma levels of CC-11050 at various time points up to 24 h in uninfected mouse treated with various concentrations (B). [file image_1.tif]

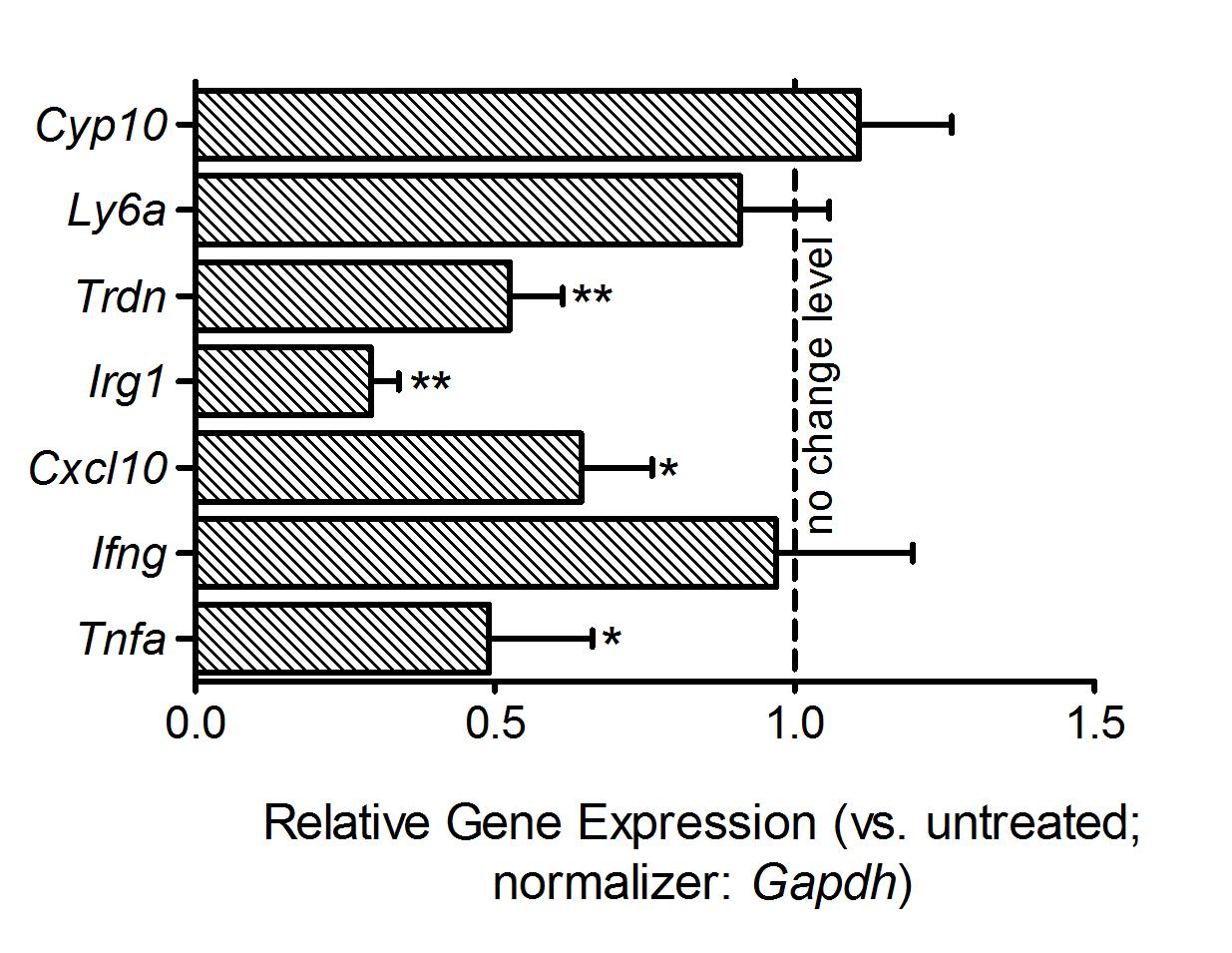

Supplement: Figure S2 — Validation of microarray data by qPCR. Randomly selected mouse genes from microarray data at 28 days postinfection was evaluated by qPCR. Values shown are expression levels in CC-11050-treated mouse lungs, compared to the untreated. Level of Gapdh expression was used to normalize the data. The experiment was repeated in duplicate with four samples per group. *P < 0.05; **P < 0.001. [file image_2.tif]

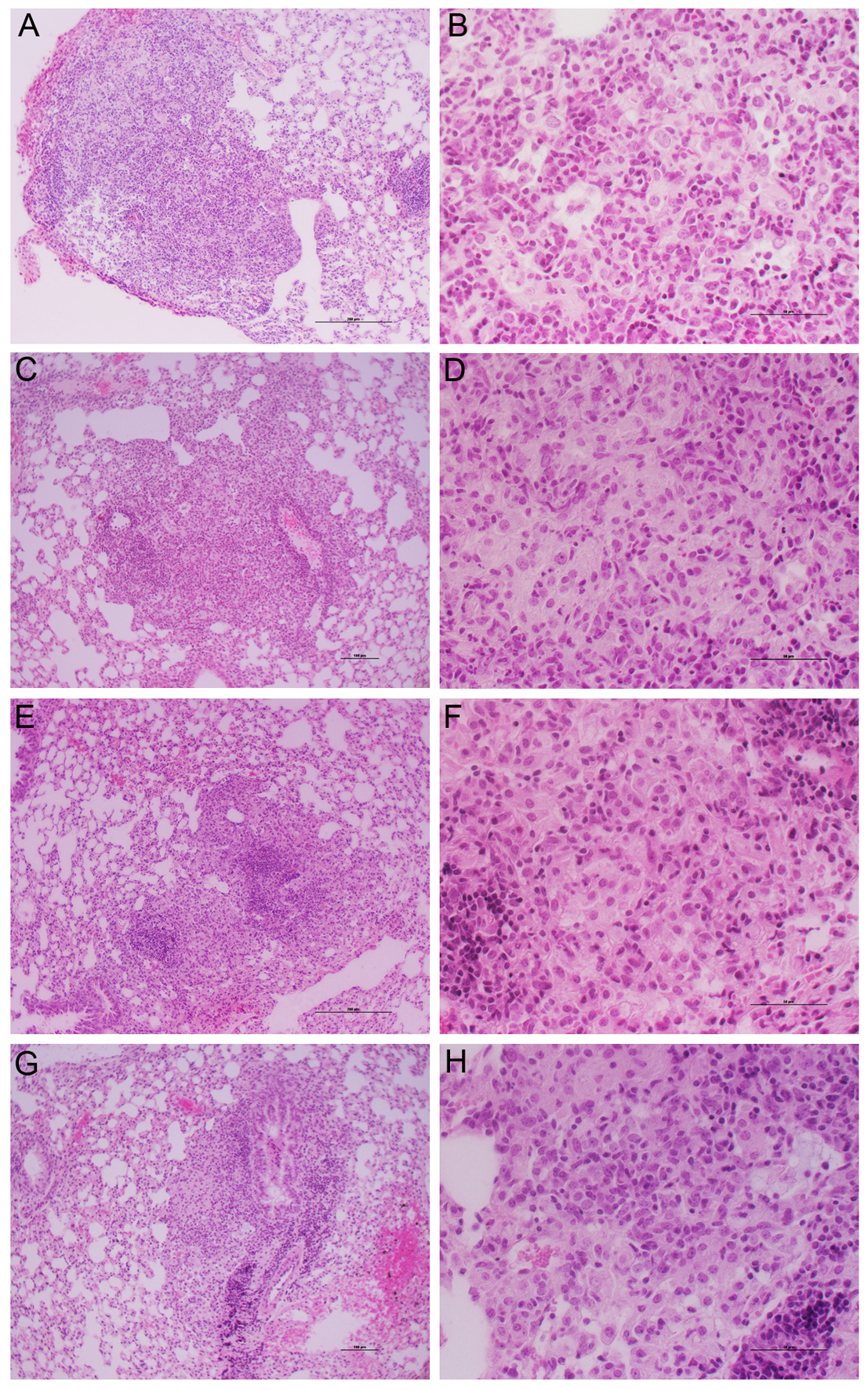

Supplement: Figure S3 — Histology of mouse lung at 28 days postinfection. Representative images of mouse lung sections at 28 days postinfection (14 days posttreatment), stained with H & E, showing the architecture of granulomas with diffuse accumulation of inflammatory/immune cells. (A,B) untreated; (C,D) CC-11050-treated; (E,F) INH-treated; (G,H) INH plus CC-11050-treated. (A,C,E,G) are photographed at 10×, and (B,D,F,H) are photographed at 40× magnification. Note that the granulomas in INH or INH plus CC-11050-treated mouse lungs are smaller than the untreated or those treated with CC-11050 alone. [file image_3.tif]
